# Is There a Mitochondrial Protection via Remote Ischemic Conditioning in Settings of Anticancer Therapy Cardiotoxicity?

**DOI:** 10.1007/s11897-024-00658-w

**Published:** 2024-03-21

**Authors:** Petra Kleinbongard, Ioanna Andreadou

**Affiliations:** 1 Institute for Pathophysiology, West German Heart and Vascular Center, University of Essen Medical School, Essen, Germany; 2 Laboratory of Pharmacology, Faculty of Pharmacy, National and Kapodistrian University of Athens, Athens, Greece

**Keywords:** Anthracycline, Cancer-treatment, Cardioprotection, Cardiotoxicity, Mitochondria, Remote ischemic conditioning

## Abstract

**Purpose of Review:**

To provide an overview of (a) protective effects on mitochondria induced by remote ischemic conditioning (RIC) and (b) mitochondrial damage caused by anticancer therapy. We then discuss the available results of studies on mitochondrial protection via RIC in anticancer therapy-induced cardiotoxicity.

**Recent Findings:**

In three experimental studies in healthy mice and pigs, there was a RIC-mediated protection against anthracycline-induced cardiotoxicity and there was some evidence of improved mitochondrial function with RIC. The RIC-mediated protection was not confirmed in the two available studies in cancer patients. In adult cancer patients, RIC was associated with an adverse outcome. There are no data on mitochondrial function in cancer patients.

**Summary:**

Studies in tumor-bearing animals are needed to determine whether RIC does not interfere with the anticancer properties of the drugs and whether RIC actually improves mitochondrial function, ultimately resulting in improved cardiac function.

## Introduction

Mitochondria are critical elements for cardiomyocyte function and viability [1, 2], and preservation of mitochondrial function is relevant for cellular homeostasis not only in normal but also in transformed malignant cells [3•]. Without any doubt, the main function of the mitochondria is the generation of the high energy phosphate carrier adenosine-5′-triphosphate via the oxidative phosphorylation. However, mitochondria are also decisive elements in the synthesis of metabolic precursors; they contribute to different cellular functions such as calcium homeostasis, generation of reactive oxygen species (ROS), immune signaling, mitophagy, and apoptosis [4]. There is a complex pathophysiological relationship between cardiovascular diseases (CVDs) and cancer, and mitochondria play a significant role [5•]. On one hand, the progressive decline of mitochondrial function occurring during CVDs is associated with alterations in the respiratory chain and adenosine triphosphate (ATP) synthesis, excessive ROS production, and structural abnormality. These processes lead to cell damage and cardiomyocyte death occurring via apoptosis, triggered by cytochrome c release, or necrosis, induced by mitochondrial permeability transition pore (mPTP) opening [6]. On the other hand, malignant transformation associated with cancer development has been associated with abnormal mitochondrial dynamics [7]. For example, mitochondrial fission mediated by unbalanced ROS production is a major cause of hepatocellular carcinoma cell survival [8] and promotes chemotherapy resistance in numerous cancers [9]. Mitophagy plays multiple roles in cancer development and progression [10]. It can serve as a tumor suppressor, maintaining the balance between mitochondria amount/activity by removing damaged or dysfunctional mitochondria in certain cancer subtypes [11]; it can reduce ROS formation and limit tumor-initiating step mediated by ROS, but can also drive drug resistance, preventing chemotherapy-induced apoptosis during tumor progression [12]. Anticancer therapy-induced mitochondrial damage drives myocardial dysfunction, resulting in several heart diseases including cardiac hypertrophy, myocardial infarction, and metabolic cardiomyopathies [13]. Therefore, cardioprotective approaches, which are known to preserve mitochondrial morphology and function in the setting of acute myocardial infarction, appear at first glance to be an interesting strategy to reduce myocardial damage during anticancer therapy [14]. Remote ischemic conditioning (RIC), short periods of ischemia/reperfusion (I/R) to tissues distant from the heart, such as the limbs, is a self-defense response that initiates systemic protection and protects a variety of organs, including the heart. RIC is one of the cardioprotective interventions which has been successfully translated from experimental approaches to the patient [15–17].

In this review, we first provide an overview of the mitochondrial protective effects induced by RIC and then review the mitochondrial damage caused by anticancer therapy, focusing on those anticancer agents whose cardiotoxicity can be prevented by RIC. Finally, we critically discuss the available results of studies on mitochondrial protection by RIC in cardiotoxicity in cancer therapy and add our personal thoughts and perspectives.

## Remote Ischemic Conditioning and Mitochondrial Protection

The interest in and knowledge of cardioprotective strategies is driven by the fact that there is substantial room for improvement in the treatment of patients with acute myocardial infarction. The gold standard for salvaging myocardial tissue during myocardial infarction is to restore blood flow to the ischemic zone of the myocardium as quickly as possible. However, reperfusion, which terminates ischemia, can also induce damage called reperfusion injury. There is currently no effective therapy for this I/R injury. The development of cardioprotective strategies to reduce I/R damage is therefore of interest [17]. Such cardioprotective strategies focus on preserving mitochondrial function; the extent of mitochondrial damage is a critical determinant of myocardial I/R injury [18]. RIC is one of the most robust mechanical cardioprotective interventions which were translated from experimental approaches to the patient [15–17]: In single-center trials on patients undergoing elective surgical coronary revascularization, RIC provided perioperative myocardial protection (e.g., [19–24]) and improved patient prognosis [23, 25]. Two prospectively designed multicenter phase III trials in patients undergoing elective surgical coronary revascularization and valve surgery, i.e., ERICCA and RIPHEART, however, were neutral [26, 27], possibly because use of propofol rather than volatile anesthesia [28]. Similarly, in patients with acute myocardial infarction first single-center trials on RIC showed myocardial protection (e.g., [29–32]) and an improved patient prognosis [33], but the prospectively designed larger phase III multi-center follow-up CONDI-2/ERIC-PPCI trial was neutral on myocardial injury and clinical outcome [34]. One prospectively designed single-center RIC-STEMI trial reported an improved clinical outcome as a primary endpoint with RIC [35]. In addition to propofol, many other factors are discussed that may reduce the robustness of the protection provided by RIC in patients [36–38]. In addition to such confounding factors, errors in the planning and design of preclinical and clinical studies are considered [17, 39, 40]. Based on some of these considerations, two clinical trials are currently underway, the Remote Ischemic Conditioning With Local Ischemic Postconditioning in High-Risk ST-elevation Myocardial Infarction (RIP-HIGH; NCT04844931) trial and the Remote Ischaemic Conditioning in STEMI Patients in Sub-Saharan AFRICA (RIC-AFRICA; NCT04813159) trial, which, in contrast to the previous trials, specifically include patients who urgently require a cardioprotective treatment in addition to standard care. The following reviews provide a comprehensive and detailed overview and critical discussion of all studies that have investigated the cardioprotective effects of RIC [16, 17].

Signal transduction from distant tissues to the heart is complex, as is signaling within the myocardium itself; however, mitochondria are the intracellular target organelle for the cardioprotective effect of RIC. RIC’s signal transfer from remote tissue to the heart is a concert of neuronal and humoral signaling [41], and the spleen seems to play a key role [42]. There is a RIC-dependent release of humoral cardioprotective factors from the spleen upon vagal activation, which is causally involved in RIC-induced infarct size reduction [43, 44]. The underlying myocardial signaling can be conceptually classified according to their (sub-)cellular localization [16, 41, 45]. Extracellular molecules, e.g., autacoids and neurohormones (acetylcholine, opioids) or ROS such as nitric oxide, are involved [41]. Such extracellular molecules then activate via sarcolemmal receptors or receptor-independent cytosolic signaling cascades. Currently, three major intracellular signaling pathways are described: the endothelial nitric oxide synthase/protein kinase G (eNOS/PKG) pathway, the reperfusion injury salvage kinase (RISK) pathway, and the survivor activating factor enhancement (SAFE) pathway [41]. The intracellular signaling pathways interact and converge on the mitochondria and modify their function [45–49] (recently discussed in detail by Yellon et al. [50] and Kleinbongard et al. [51]).

Downstream of the intracellular signaling, activated by RIC, mitochondrial function is modified as follows: I/R causes damage to the *mitochondrial respiratory chain*, which in a physiological state transfers electrons from Krebs cycle substrates to oxygen and then creates a protein gradient across the inner membrane. This gradient results in a hyperpolarization, which then drives mitochondrial *ATP formation* [46]. In mitochondria, isolated from mouse [52] and rat hearts [53] at early reperfusion, the I/R-mediated reduction in oxygen consumption rate was increased by RIC. Mitochondrial respiration was also improved in mitochondria isolated from isolated perfused rat [43, 54] and neonatal rabbit hearts [55] after perfusion with plasma or its derivatives from conditioned rabbits [55] or pigs [43, 54]. The RIC-dependent improved mitochondrial respiration and ATP production was not only demonstrated at early reperfusion but also up to 14 days of reperfusion in rats without [56] as well as in rats with streptozotocin-induced diabetes [57]. In a pig model with acute I/R, exenatide or glucose-insulin-potassium treatment in addition to RIC induced additive cardioprotective effects and further improved mitochondrial function at early reperfusion [58]. RIC improves the mitochondrial respiration not only in animal models but also in patients: With RIC, mitochondrial respiration was improved in left ventricular biopsies [59] and right atrial appendages [60, 61] of patients undergoing cardiac surgery. Mitochondrial telomerase reverse transcriptase (TERT), which improves complex I subunit composition, seems to be critical for improved mitochondrial respiration with RIC in the human heart mitochondria [62]. Associated with the increased mitochondrial respiration, mitochondrial ATP production was increased with RIC in animal models and in patient myocardium [43, 54, 61]. In isolated trabecula from human right atrial tissue, improved mitochondrial function went along with improved contractile function and was associated with myocardial protection by assessed from serum troponin I / troponin T release in the patients after cardiac surgery [61]. Disturbed electron flow during ischemia leads to increased *generation of ROS* [63]. This increase in ROS formation after I/R was also reduced by RIC, which has been demonstrated not only in the various above described animal models [43, 52, 54] but also in human tissue [61]. One study in rats identified an increase in mitochondrial ROS formation direct after RIC, but before I/R [64]. In fact, the higher ROS production prior to myocardial ischemia may protect the heart from the increased ROS production during I/R [63]. The *mPTP* is mostly closed during physiological conditions. During ischemia, e.g., in response to increased ROS and calcium, mPTP opens, which results in cation flux into the mitochondria and consequently mitochondrial swelling and eventual rupture of the mitochondrial outer membrane. MPTP opening is enhanced during early reperfusion when the inhibitory effect of acidosis is reversed, ultimately leading to cardiomyocyte death [65]. RIC was associated with an inhibition of mPTP opening at reperfusion, again demonstrated not only in the various above described animal models [43] but also in human tissue [61]. Not only the mPTP but also other *mitochondrial ion channels* control the potential of the inner mitochondrial membrane, the matrix volume, respiratory chain function, calcium homeostasis, and ROS formation [63]. In rats, opening of the mitochondrial K_Ca_ channel contributes to RIC-induced cardioprotection by maintaining mitochondrial manganese superoxide dismutase and mitochondrial membrane potential [66]. Mitochondria are dynamic organelles, which undergo *fission* (division of a mitochondrium) or *fusion* (merging of outer and inner mitochondrial membranes) in order to maintain mitochondrial form and integrity. During myocardial I/R, mitochondria become fragmented, and consequently, the inhibition of mitochondrial fission preserves mitochondrial and myocardial function and reduces cell death [48]. RIC in rats not only decreased infarct size but also increased mitochondrial fusion protein optic atrophy-1 and preserved mitochondrial morphology [67]. In isolated rat, heart mitochondria RIC also resulted in an elimination of I/R-induced reduction of *mitochondrial membrane fluidity* [53]. Perturbations in mitochondrial morphology, i.e., activation of fusion confers cardioprotection, seem to comprise delayed mPTP opening and decreased ROS formation. Mitochondrial fission precedes *mitophagy*, a process by which damaged mitochondria are removed and which represents a part of autophagy (for review, see [48]). However, depending on the model and the duration of I/R, both an activation and an inhibition of mitophagy are described (for review, see [68]). Consequently, the activation of mitophagy may exert protective effects, whereas a marked induction of the process may be detrimental in the context of I/R injury. The cardioprotective effects are mediated by the activation of autophagy [68].

## Mitochondrial Damage Caused by Chemotherapeutics and Targeted Therapy

Currently, we have a wide portfolio of available anticancer therapies; since they are administered systemically, the mitochondria are also affected systemically, and since the heart has an insane number of mitochondria that are needed to maintain cardiac function, the unwanted side effects here are particularly large—the so-called establishment of cardiotoxicity. Although the cardiotoxic side effects are categorized on a class effect basis, mitochondrial targets are major determinants of the cardiotoxic effects triggered by an increasing number of anticancer drugs [69]. Recent reviews [3•, 5•] are very comprehensive and take into account the various effects of different anticancer agents on the mitochondria; therefore, we have focused here on those that can possibly be altered by RIC.

### Chemotherapeutics

The ability of the *anthracycline doxorubicin* to accumulate primarily in mitochondria may therefore explain the cardio-selective toxicity of the drug. At the molecular level, doxorubicin enters mitochondria (because of to its cationic nature) and accumulates within the inner membrane, where it binds to the cardiolipin [70]. Cardiolipin plays a role in mitochondrial membrane structure and regulates the activity and function of mitochondrial proteins, including the enzyme complexes of the electron transport chain. Consequently, with doxorubicin, *mitochondrial respiration* and the *production of ATP* are reduced, and *ROS generation* is increased in cultured neonatal myocytes and adult rat hearts [71, 72]. Additionally, an affected reduction in mitochondrial mass is observed upon treatment with doxorubicin, which activates mitophagy in human adult ventricular cardiomyocytes [73] and inhibits mitochondrial biogenesis in rabbit hearts [74]. Subsequently, ROS accumulation within mitochondria results to *mPTP* opening and apoptosis [71], further contributing to cardiomyocytes loss. Additionally, doxorubicin has been shown to stimulate the receptor-interacting protein 3-induced activation of Ca^2+^-calmodulin-dependent protein kinase, triggering mPTP opening, thus inducing myocardial necroptosis in mice [75]. Therefore, doxorubicin can dose dependently stimulate mPTP, because of mitochondrial oxidative damage and Ca^2+^ overload since are both potent inducers of mPTP opening involved in its cardiotoxic effects [76]. Preclinical and human studies indicate that mitochondrial impairment, redox, and metabolic alterations persist after doxorubicin therapy completion and that the toxic effects of doxorubicin can lead to cumulative dose-dependent and progressive mitochondrial dysfunction correlating with the drug’s cardiotoxicity memory [77]. Besides, there is also growing evidence that anthracyclines can disrupt *mitochondrial dynamics*, which is increasingly recognized as a major process driving anthracyclines’ cardiotoxicity. In vitro and in vivo data indicate that doxorubicin shows inhibitory effects on mitochondrial fusion while promoting mitochondrial fission. More specifically, increased dynamin-related protein 1 expression protein levels that have been found in patients with ischemic cardiomyopathy and dilated cardiomyopathy [78] represent a key factor also promoting the shift towards mitochondrial fission during doxorubicin exposure [69].

Among the *alkylating agents*, high doses of cyclophosphamide and ifosfamide may induce acute myopericarditis and severe arrhythmias, which have been shown to be associated with impairment of mitochondrial transport and oxidation of long chain fatty acids in rats [79], abnormal *mitochondrial respiration* as well with as increase of *ROS* [80]. Cancer cell lines, that respond to cisplatin, have greater levels of mitochondrial ROS than cisplatin-resistant cancer cells [81], and cisplatin affects mitochondrial DNA and increases mitochondrial ROS production in cancer cells; mechanisms involved in its cytotoxic effects [82].

As far as *antimetabolites* are concerned, among the severe side effects of 5-fluorouracil (5-FU) is also cardiotoxic [83] through vascular dysfunction with microthrombi formation [83]. 5-FU induces mitochondrial dysfunction characterized by *low ATP levels*, loss of *mitochondrial membrane potential*, and excessive mitophagy ending in *mitochondria-mediated apoptosis* in freshly isolated rat cardiomyocytes [84]. Furthermore, the accumulation of the highly toxic intermediate fluoroacetate from the biotransformation of 5-FU may influence mitochondrial function by inhibiting the Krebs cycle [85].

In summary, although anthracyclines have been extensively studied regarding their cardiotoxic effects in which mitochondrial dysfunction plays an important role, alkylating agents and antimetabolites have been correlated with cardiotoxic effects mediated by mitochondria. Thus, RIC may be an important interventional approach to reduce the cardiotoxic effects of chemotherapeutic agents.

### Targeted Therapies

Among the *tyrosine kinase inhibitors*, imatinib and sunitinib are two representative examples of on-target [86] and off-target [87] cardiotoxicity, respectively. The mechanism includes mitochondrial dysfunction, uncoupled *mitochondrial respiration*, mitochondrial Ca^2+^ overload, and increased *ROS generation* [88]. Studies have shown that sunitinib affects mitochondrial function through inhibition of AMP-activated protein kinase initiating changes in adenosine monophosphate (AMP):ATP ratio, a central regulator of energy/mitochondrial homeostasis [89], and both in cardiac cells and in vivo sunitinib inhibited mitochondrial β oxidation, impaired electron transport chain activity, and increased mitochondrial ROS production [90–92]. Analysis of endomyocardial biopsies obtained from patients who developed chronic heart failure after treatment with imatinib mesylate revealed significant ultrastructural mitochondrial changes and abnormalities [93]. Additionally, *oxidative phosphorylation* resulting in uncertain *ATP production* and deranged *mitochondrial energetics* were also observed in response to clinically relevant concentrations of sorafenib in isolated rat heart mitochondria and H9c2 cells [94, 95].

The cardiotoxic effects of *trastuzumab* are in part mediated by its action of blocking the function of the human epidermal growth factor receptors (HER) 2 ligand neuregulin. Trastuzumab results in activation of mitochondria-mediated apoptosis in combination with mitochondrial respiratory dysfunction, *reduced ATP levels*, impaired redox capacity, and loss of mitochondrial *membrane potential* in cardiomyocytes [96]. Trastuzumab by inhibiting neuregulin function initiates the harmful effects of *oxidative stress*, leading to DNA breakage and induction of *mitochondrial apoptosis* [97]. Trastuzumab alters the balance of pro- and antiapoptotic B-cell lymphoma-2 proteins, which promotes mitochondrial release of cytochrome-c and initiates caspase activation and apoptosis in cardiomyocytes [96].

Data obtained from animal models and from patients demonstrate that proteasomal inefficiency, together with increased levels of protein ubiquitination, correlates with cardiomyopathies [98]. Multiple myeloma accounts for 1% of neoplastic diseases and *proteasome inhibitors* (PIs) stand as an important anti-myeloma therapy [99]. The cardiotoxicity of the reversible PI, *bortezomib*, is still under debate, and possibly depends on whether the drug is administered in patients with cardiovascular disease risk factors [100], but the irreversible PI, carfilzomib, has been associated with severe cardiac adverse effects [101]. Mitochondria have been identified as a relevant target of cardiotoxicity because bortezomib *inhibits complex V of the respiratory chain*, resulting in *reduced ATP* synthesis in rat hearts and in a decreased cell shortening of primary rat left ventricular myocytes [102]. Carfilzomib treatment in human-induced pluripotent stem cell-derived cardiomyocytes reduced *mitochondrial membrane potential*, *ATP level*, and *mitochondrial oxidative respiration* [103]. Since the mitochondrial *fission and fusion* proteins are recycled by the ubiquitin–proteasome system, regulation of the mitochondrial dynamics may be affected by proteasome inhibition [104], and in the above-mentioned model, carfilzomib resulted in downregulated expression of mitochondrial fusion proteins (mitofusin 1 and optic atrophy 1) [103].

In summary, the cardiotoxicity induced by targeted therapies is also mostly related to alteration of mitochondrial dynamics, reduced ATP levels, and impaired redox capacity, all of which can again theoretically be abrogated by RIC.

## Remote Ischemic Conditioning and Mitochondrial Protection in Settings of Anticancer Therapy Cardiotoxicity

Although it seems obvious that RIC could also protect against cardiotoxicity—at least the targets at the mitochondrial level seem to be operable by RIC—the idea of utilizing the cardioprotective effect of RIC in the field of anticancer therapy cardiotoxicity is relatively new and has not yet been thoroughly investigated. Just five studies have focused on RIC in anticancer therapy cardiotoxicity—two experimental studies in mice [105•, 106•], one in the large animal model pig [107•], and two proof-of-concept clinical studies, one in pediatric cancer patients [108•] and the other in adult patients [109•]. All preclinical studies focus on cardiotoxicity caused by the anthracyclines, but mitochondrial function has only been investigated in two experimental studies [105•, 107•].

In the experimental studies, all of the animal models without cancer, cardiotoxicity of anthracyclines (whether administered i.p. [105•, 106•] or i.c. [107•] and independent which dose was administered) has been documented to varying extends, but a reduction in left ventricular function has been demonstrated in both the mouse [106•] and the pig model [107•]. RIC reversed anthracycline-induced cardiotoxicity in all animal models by not only improving left ventricular function [107•] but also reducing acute myocardial injury [106•], myocardial inflammation [106•], fibrosis [105•, 106•, 107•], and apoptosis [105•], enhancing autophagy [105•] and improving mortality over an 85-day observation period [105•] (Table 1). In agreement with the concept that RIC induces systemic effects, the cytoprotective effects were not limited to the heart; organ weight and function of the kidney, spleen, and liver were also preserved in one mouse study [106•].

**Table 1:** Overview of all available studies on anticancer therapy cardiotoxicity and protection from it via RIC

Species	Cancer	Anticancer drug	RIC procedure and timing	Group *number*	Major findings on cardiotoxicity	Major findings on cardioprotection	Effects on mitochondria
CD1 mice, adult [105•]	Without	Doxorubicin, 1 × 20 mg/kg, i.p	One limb, 3 cycles of 5 min ischemia/reperfusion 1 h before doxorubicin	Doxorubicin/doxorubin + RIC *n* = *43/43* for 85 days *n* = *25/25* for 25 days	n.d	⇓ mortality over 85 days ⇑ left ventricular mass ⇓ fibrosis and apoptosis ⇑ autophagy at day 25	⇔ mitochondrial oxidative phosphorylation at day 25
C57 mice, adult [106•]	Without	Doxorubicin 1 × 10 mg/kg, i.p	One limb, 4 cycles of 5 min ischemia/reperfusion before doxorubicin starting 30 min before doxorubicin daily for 5 days	Saline/doxorubicin/doxorubin + RIC *n* = 8/8/8	⇓ left ventricular ejection fraction at day 6	⇓ myocardial injury ⇓ myocardial inflammation ⇓ myocardial fibrosis ⇑ autophagy at day 6	n.d
Large white pigs, juvenile [107•]	Without	Doxorubicin 5 × 0.45 mg/kg i.c. injections at week 0, 2, 4, 6, 8	One limb, 3 cycles of 5 min ischemia/reperfusion repetitively immediately before doxorubicin	Without doxorubicin/doxorubicin/doxorubin + RIC *n* = *10/10/10*	⇓ left ventricular ejection fraction ⇑ mitochondrial fragmentation at 16 weeks	⇑ left ventricular ejection fraction ⇓ fibrosis at 16 weeks	⇓ mitochondrial fragmentation at 16 weeks
		Doxorubicin 3 × 0.45 mg/kg i.c. injections	Single limb, 3 cycles of 5 min ischemia/reperfusion immediately before doxorubicin	Doxorubicin/doxorubin + RIC *n* = *5/5*	n.d	⇔ left ventricular ejection fraction at 6 weeks	⇓ mitochondrial fragmentation and morphological abnormalities ⇓ mitochondrial fission ⇓ autophagy at 6 weeks
Human, pediatric patients [108•]	Solid tumors, hematologic malignancies	Respective anthracycline regimens for the different types of malignancies cumulative dose: ~ 155 mg/m^2^	One upper or lower limb, 3 cycles of 5 min ischemia/reperfusion within 24 h after each anthracycline treatment with a maximum of 4 times per patient	Anthracycline + sham/anthracycline + RIC *n* = *34/34*	⇑ myocardial injury ⇓ left ventricular function over 1 week and at 3 months	⇔ myocardial injury ⇔ left ventricular function over 1 week and at 3 months	n.d
Human, adult patients [109•]	Any cancer, designated to anthracycline-therapy	Doxorubicin cumulative dose: ~ 315 mg/m^2^	One limb, 4 cycles of 5 min ischemia/reperfusion before each doxorubicin treatment 2–6 times per patient	Doxorubicin + sham/doxorubicin + RIC *n* = *27/28*	⇑ myocardial injury ⇓ left ventricular function over 1 year	⇔ myocardial injury ⇔ left ventricular function ⇓ 1 year outcome (increased number of major adverse cardiac events or cancer death)	n.d

*i.c.* intracoronary, *i.p.* intraperitoneal, *n.d.* not determined, *RIC* remote ischemic conditioning

Although no experimental studies on the effect of RIC in tumor-bearing animals exist, clinical trials have been initiated. Neither the study in pediatric cancer patients [108•] nor in that in adult patients [109•] reproduced the cardioprotective effects observed in animal models— no effects on left ventricular function or acute myocardial injury (Table 1). More importantly, in the trial with adult patients [109•], there was a significant increase in combined major adverse cardiovascular events and cancer death with RIC (11 versus 3) but also in cancer death alone (8 versus 1) after 1-year follow-up. There is one ongoing trial, the REmote iSchemic condItioning in Lymphoma PatIents REceiving ANthraCyclinEs (RESILIENCE) NCT05223413, a proof of concept phase II trial with the aim to evaluate the efficacy and safety of RIC in lymphoma patients receiving anthracyclines. Endpoints are acute myocardial injury and left ventricular function over a median follow-up of 24 months.

It is important to state herein that all the studies with RIC have been conducted to investigate its beneficial effects against only doxorubicin-induced cardiotoxicity; however, targeted therapies have also shown important cardiotoxicity. Recent therapies targeting the immune system rather than the tumor cells only represent a feasible and successful therapeutic strategy. Immune-checkpoint inhibitors (ICIs) exhibit remarkable anti-tumor activity, but they also exhibit significant cardiotoxicity, and there is an unmet clinical need for efficient management of ICI-related cardiovascular adverse effects [110]. Although the mechanisms implicated in the cardiotoxicity of ICIs have not been elucidated, mitochondrial impairment may also characterize a key effector of ICI-induced cardiotoxicity [3•].

## Thoughts and Perspectives

### Are these disappointing or even alarming results in the human studies completely unexpected? What is the difference between the experimental studies in animals and the clinical studies?

The same cardiotoxic medication was investigated (in both cases anthracyclines). The animals are rather juvenile or young compared to patients—although one of the clinical trials was in pediatric patients. Compared to the adult patients, the animals do not have comedications and comorbidities, which are often discussed as confounding factors for cardioprotection by RIC [37, 38, 111]. However, neither comedications nor comorbidities seem to be responsible for the worse outcome with RIC in adult patients. Thus, is the key difference that no animal model has looked at the effect of RIC on the tumor growth. Again, all the animal studies have been done in healthy animals. Studies on RIC in the different clinical settings (in patients with stroke, acute myocardial infarction, or cardiac surgery), to the best of our knowledge did not report an increase in tumor disease. Also, in patients undergoing colorectal cancer resection without pharmacological cancer therapy, RIC improved postoperative gastrointestinal dysfunction, and no effect on tumor growth was described [112]. However, RIC is associated with blockade of splenic immune cell release, including T cell, B cell, and NK cell, into circulation (for review, please see [113])—and T cells as well as NK cells are relevant for tumor growth [114, 115].

**Fig. 1:**
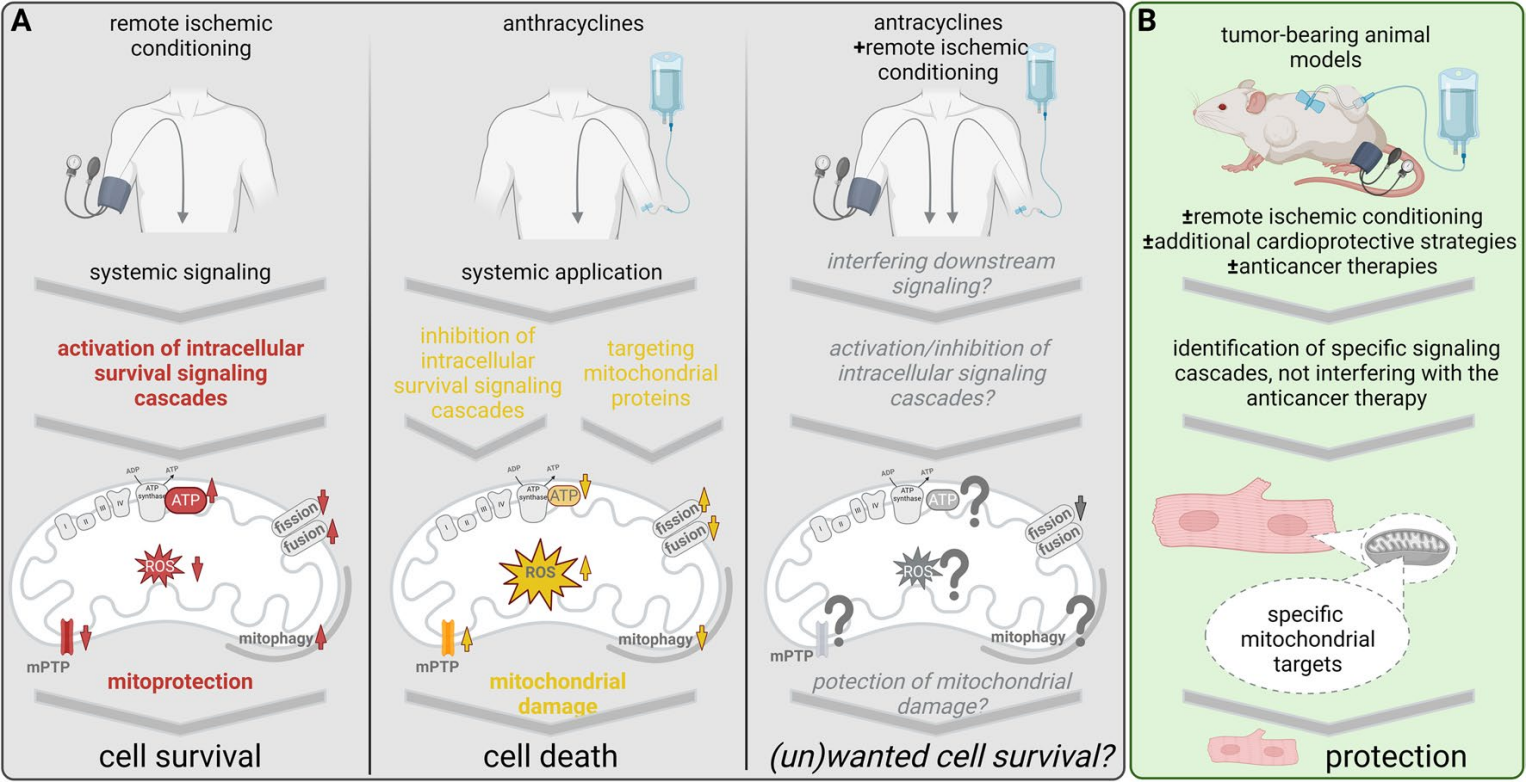
**A** Overview of known effects of remote ischemic conditioning and anthracyclines on mitochondrial function: remote ischemic conditioning activates intracellular survival signaling cascades all of them converge to mitochondria resulting in mitochondrial protection, cell survival, and thus, cardioprotection. Anthracyclines induce mitochondrial dysfunction and induce cell death and as side effect cardiotoxicity. Currently, it is unclear, whether remote ischemic conditioning really prevents the anthracycline-induced mitochondrial dysfunction and thus cell death, or whether it is even involved in an unwanted cell survival. **B** Consequently, there is a need for studies in tumor bearing animals in order to clarify the effects of remote ischemic conditioning and anticancer therapies on mitochondrial function and finally on protection against anticancer therapy cardiotoxicity. I, II, II, IV, mitochondrial respiratory chain complexes; ADP, adenosine diphosphate; ATP, adenosine triphosphate; mPTP, mitochondrial permeability transition pore; ROS, reactive oxygen species. Created with Biorender.com

Therefore, as step one, we need studies to be conducted in animals—and at best in animal models which are the closest to humans [116•]—with tumors in order to clarify if RIC interferes with tumor growth and if the cardioprotective effects of RIC do not hamper the anticancer properties of the drugs and importantly to check for both therapy success and reduction of anticancer therapy cardiotoxicity (Fig. 1). As step two, we need studies to be conducted with different anticancer therapies since although we have many common signaling mechanisms in mitochondria, each agent may differentially affect ATP, ROS, mPTP, and/or mitochondrial dynamics, the main signaling pathways that have been found so far important for the cardiotoxicity induced by different anticancer medications (Fig. 1).

### What do we know about intracellular signaling in cardiotoxicity targeting mitochondria?

The studied effects on mitochondria, so far investigated, appear to be comparable to those described for I/R. In pig hearts, RIC reduces mitochondrial fragmentation caused by anticancer therapy cardiotoxicity [107]. These effects on mitochondria were evident at both 6 and 16 weeks after the start of doxorubicin, although the improvement in left ventricular function in the RIC group was not seen until 16 weeks (Table 1). Mitochondrial oxidative phosphorylation was studied in the mouse model, but was unchanged 25 days after doxorubicin treatment [105]. It may have been too early to quantify the effect on mitochondrial function, the cardioprotective effect of RIC has already been demonstrated in animals in the form of a reduction in fibrosis and apoptosis, an increase in left ventricular mass and increased autophagy [105] (Table 1). Overall, therefore, there is just little evidence from the two animal studies suggesting an effect of RIC on cardiac mitochondria. Whether this is transferable to humans and whether this effect is limited to cardiac mitochondria or is a systemic effect on mitochondria remain unclear. RIC, however, acts systemically, so it must be assumed that mitochondrial function is influenced systemically—thus also those of tumor cells.

### Do the two systemic approaches “RIC” and “anticancer therapy” target the opposite?

RIC activates cell-protective intracellular pathways, with the intracellular target mitochondria and associated with cell survival whereas cancer therapy also targets the intracellular endpoint mitochondria, but with the aim of inducing cell death. The main signaling molecules of the major cardioprotective intracellular signaling pathways, the eNOS/PKG, the RISK, and the SAFE pathways [16, 41, 45] constitute also targets of anticancer therapy [117] (Fig. 1). One key protein of the cardioprotective SAFE pathway, the transcription factor STAT3, improved mitochondrial function when activated acutely in settings of I/R [51]. However, repetitive RIC—as done in most studies related to anticancer therapy cardiotoxicity (Table 1)—may activate systemically the transcription factor and may consequently foster malignant transformation and promote cancer [118, 119]. However, whether there is an interaction between activation of intracellular signaling cascades via RIC, cancer/anticancer therapy is currently not known and needs further clarification (Fig. 1). Same is true on mitochondrial level, i.e., cardioprotection is associated with mitochondrial TERT which improves mitochondrial function in animal models and in patient myocardium [62]. Since TERT has been also shown to be a regulator of mitochondrial ROS [120], recent in vitro studies in human-induced pluripotent stem cell-derived cardiomyocytes presented anti-apoptotic effects of telomerase overexpression after doxorubicin treatment, whereas overexpression of telomerase protected the heart from doxorubicin-mediated apoptosis in a mouse model of chronic doxorubicin-induced cardiotoxicity [121]. Therefore, it might be of outmost importance to focus on new signaling molecules in mitochondria in order to unravel molecular mechanisms and novel targets of cardioprotection (Fig. 1).

### Potential preventive strategies focused on mitochondria for anticancer therapy cardiotoxicity

A recent meta-analysis focused on the efficacy of beta-blockers or angiotensin-converting enzyme inhibitors/angiotensin II receptor blockers for prevention of cardiotoxicity concluded that the beneficial effects of the above-mentioned drug categories throughout the studies were variable as documented by significant heterogeneity between the studies, and systematic evidence is needed to recommend drugs for cardioprotective prevention during chemotherapy [121]. Cardio-oncology provides a chance for more precision-based strategies that require a vast identification of the underlying molecular mechanisms [117]. Strategies that converge to mitochondria (such as RIC) combined with strategies that act on a different cell population or different signaling molecules maybe an ideal alternate that would be useful as an effective treatment approach. However, it is essential to first understand the individual intracellular and mitochondrial signals involved (Fig. 1).

## Conclusion

RIC activates intracellular survival signaling cascades; all of them converge to mitochondria resulting in mitochondrial protection and subsequently cardioprotection. Current therapy of advanced cancers is based on several modalities, all can negatively impact the cardiovascular system, and there is considerable experience in relation to radiotherapy and chemotherapy [13]. Anthracyclines have been extensively studied regarding their cardiotoxic effects in which mitochondrial dysfunction plays an important role and the key common cardiotoxic mechanisms include mitochondria-mediated apoptosis, mitochondrial ROS production, and mitophagy. Although RIC could play an important role in preventing anthracycline-induced cardiotoxicity and the very few preclinical studies showed positive results, this failed to be translated in the clinical arena, so far. Additional studies are necessary especially in tumor bearing animals in order to clarify the (i) effects of RIC, (ii) of anticancer therapies combined with RIC and shed a light for potential combinational therapies with RIC as important strategies for prevention of anticancer therapy cardiotoxicity (Fig. 1). These studies should also identify new specific mitochondrial targets not interfering with the anticancer therapy and investigate the role of RIC as an important or maybe not interventional approach to reduce the cardiotoxic effects of anticancer agents. Conventional cytotoxic chemotherapy and targeted and immune therapies are the most effective treatment options for many types of cancer. However, cardiotoxicity, especially the decrease in left ventricular function with these therapies, impairs prognosis; thus, prevention and treatment of cardiotoxicity are crucial [122].

## Data Availability

No datasets were generated or analyzed during the current study.
